# “Who generates this city”? Socialist strategy in contemporary London

**DOI:** 10.1111/1468-4446.12751

**Published:** 2020-03-30

**Authors:** Jacob Mukherjee

**Affiliations:** ^1^ Department of Media, Communications and Cultural Studies Goldsmiths University of London London United Kingdom

**Keywords:** activism, discourse, London, political identities, social movements

## Abstract

This essay, based on a “militant ethnography” of the attempts of the small radical grassroots activist group, Our London (a pseudonym), to mobilize a collective oppositional politics through activities around an election campaign, engages critically with E. Laclau and C. Mouffe's arguments on discourse and collectivity in *Hegemony and Socialist Strategy* (London: Verso, 1985). I argue, on the basis of my findings, that while their model does provide insights that help describe the process of building collectivity from among disparate perspectives and identities, we need to go beyond a focus on discourse alone and consider the ways politics is shaped by material contexts. This is necessary if we are to understand the continued appeal of class politics as well as the difficulties in mobilizing collectivity in highly unequal and fragmented cities. From an activist perspective, the essay also highlights how developing a conception of collective interests and a critique of overarching systems of exploitation can be important in building political unity.

## 1 INTRODUCTION

This essay addresses a central question within both political organizing and the study of the contemporary grassroots Left: how might a unified, collective, radical oppositional politics develop within an urban context characterized by the “individualization of labor” (Castells, 1996, p. 265), weakening class identification, and the erosion and hollowing out of social democratic institutions and cultures over the last 40 years (Crouch, 2004)? While the issue of Left mobilization under contemporary capitalism has been much discussed in abstract terms (e.g., Dean, 2012; Laclau & Mouffe, 1985; Riofrancos, 2018), this essay draws upon rich empirical data to consider in detail the dynamics through which political unity is negotiated, contested and won at a micro level.

I draw in this essay upon my PhD research, for which I carried out a critical “militant ethnography” (Juris, 2007) aimed at investigating the attempts of the small radical grassroots activist group Our London (OL)—a pseudonym—to mobilize a collective oppositional politics within a highly unequal city. The group was small, without formal structures, and ran on very few resources. OL’s main focus during the research period was on building a program and support for a campaign for the 2016 London elections, in which the group stood a candidate. I was a founding member of the group studied, and heavily involved in its activities for much of the research period; my account is therefore necessarily a partial one informed by my own particular experiences, perspective, and interpretations.

This essay engages with Laclau and Mouffe’s (1985) argument that, rather than emerging automatically due to essential commonalities between particular subjects, political collectivity results from the articulation of those commonalities through discourse. I will show how this framework is useful in providing some of the tools to help us describe both political discourses and aspects of organizing culture on sections of the grassroots radical Left in cities like London. However, I also argue that, in dealing abstractly with the question of unity, Laclau and Mouffe—like others influenced by poststructuralist perspectives—tend to neglect both the concrete dynamics of political activity as well as the extent to which that activity is rooted in, and therefore must respond to, material realities. There is a need to emphasize, to a greater extent than Laclau and Mouffe, the material context of what those authors call “discourse.”

In respect to my case study, this theoretical framing allows me to consider the importance for the Our London project of identifying a common enemy as a way of uniting disparate perspectives and identities. I also discuss its attempts to move beyond a merely negative form of unity to develop a stronger sense of common purpose and shared social location, and suggest that both its successes and its failures in this respect must be understood in relation to the opportunities and challenges of organizing in a highly unequal and fragmented city like London. Below, I outline my theoretical framework in detail before applying it to analyze my case study.

## 2 FROM CLASS TO RADICAL DEMOCRACY? HEGEMONY AND CHAINS OF EQUIVALENCE

Laclau and Mouffe's work has proven influential, in part because it is seen to explain contemporary political conditions characterized by multiplying struggles and identifications defined in terms other than class (Fenton, 2016). However, due to their insistence on the complete “autonomization of the political” (1985, p. 31) from the economic, Laclau and Mouffe pay little attention to the materiality of capitalist exploitation with which discourse and strategy must relate. Since Laclau and Mouffe deny both that material conditions produce political subjectivity and that class should have primacy, their model cannot adequately address the significance of objective material interests. I argue—and suggest that the empirical data I present demonstrates—that a notion of collective interests can in fact have an important role in mobilizing political collectivity: that a sense of common interests can unify in a way that more abstract appeals to radical democracy, like those proposed by Laclau and Mouffe, cannot. In this section, I outline the key features of Laclau and Mouffe's model and critically discuss its benefits and shortcomings.

Laclau and Mouffe's central argument in *Hegemony and Socialist Strategy* is that “there is no logical and necessary relation between socialist objectives and the positions of social agents in the relations of production.” In other words, dynamics of capitalist exploitation and accumulation do not determine political consciousness and, therefore, any politics—socialist or otherwise—relies upon the establishment of particular identities, goals and concepts as “hegemonic” (1985, p. 86). While a number of theorists in the Marxist tradition—including, notably, Gramsci (1971)—focus on the importance of achieving hegemony, Laclau and Mouffe go further than others in denying that “class positions” are “the necessary location of historical interests” (1985, p. 85). For them, decentering class creates opportunities for socialists to engage, ally with and incorporate strands of social movement politics—from radical feminism to environmentalism—mobilized around oppressions and identities other than class; indeed, Laclau and Mouffe argue that the “hegemonic recomposition” associated with the rise of new social movements since the 1960s should prompt the socialist movement to “abandon its class ghetto and transform itself into the articulator of a multiplicity of antagonisms and demands stretching beyond itself” (1985, p. 58).

Laclau and Mouffe's model appears to present an appropriate strategic response to the contemporary conjuncture. They argue that the “new forms of struggle in the advanced capitalist countries, where during the last few decades we have witnessed the constant emergence of new forms of political subjectivity cutting across the categories of the social and economic structure” underline the need for a new model of socialism that can connect to these movements (1985, p. 13). This description of the way socio‐political identities have changed is consistent with the findings of sociologists who have pointed to factors like the expansion of higher education, economic restructuring, the rise of networked, insecure work and the emergence of increasingly individualized subjectivities as undermining earlier patterns of class identity (Beck, 2007; Savage et al., 2015; Sennett, 2006).

Another way Laclau and Mouffe's work is useful is in terms of the implications of their model for the organizational structure, culture, and processes of radical political groups. This is an aspect of their framework that has received much less attention than their ideas around class, materiality, and discourse. Laclau and Mouffe argue that coalitions of subordinate forces must, in order to advance a common struggle, keep in view “the horizon of an impossible totality” (p. 122), but be aware that “the moment of the ‘final’ suture never arrives” (p. 86), in that both collective identities and hegemonic discourses will always remain partially open. This means that political spaces—at the level of national, international, and specific local political formations—should remain forever open and contestable to allow “the multiplication of democratic struggles” (p. 131).

Such a proposal is clearly incompatible with the model of the highly centralized Leninist party that Laclau and Mouffe criticize, and much more conducive to open, inclusive, and horizontal organizing cultures. For example, Tormey counterposes the Leninist party to the social forum structures that developed out of the turn‐of‐the‐century global justice movement. Social forums were spaces characterized by “discussion, comparison, affinity,” “affiliation,” and “experimentation,” and within which no one perspective, identity, or set of interests were able to dominate or own the space (2005, p. 404). Though the social forums have largely disappeared, organizing cultures on sections of the contemporary radical Left retain similar principles. Such cultures are likely more appealing than those of the Leninist party to a contemporary activist imaginary which, as Della Porta (2005) has noted, tends to define itself in relation to concrete goals rather than fixed social, ideological, or organizational belonging and which is tolerant of a range of political perspective and identities.

While Laclau and Mouffe produce some useful insights that can be applied to analyze the contemporary activist scene, their work has some shortcomings. Chief among these is their refusal to assign central importance to the material context within which politics takes place. A key element of Laclau and Mouffe's model is its emphasis on discourse as key to mobilizing political collectivity, and their related denial that material conditions in themselves necessarily produce particular political consequences. Laclau and Mouffe affirm that there is a material reality, but that what matters from a political perspective is how discourse interprets and constructs that reality: “an earthquake or the falling of a brick is an event that certainly exists … But whether their specificity as objects is constructed in terms of ‘natural phenomena’ or ‘expressions of the wrath of God’, depends upon the structuring of a discursive field” (1985, p. 108). While this statement is designed to reassure readers that the authors are not naive idealists—that they do believe in a material reality—it in fact underlines Laclau and Mouffe's radical departure from the materialism of traditional Marxist perspectives. This is clear if we substitute “the falling of a brick” for a more politically consequential set of material realities. We might expect, for example, that an economy dominated by small yet heavily surveilled workplaces situated within cities that contain few public spaces where people can assemble, presents socialists with a very different organizing context to one characterized by large factories and numerous public squares; we might further anticipate that the routes and strategies through which workers can be mobilized and a sense of class subjectivity constructed in the two societies would differ greatly. Yet Laclau and Mouffe suggest that such realities are of less importance than, or at least possess no particular explanatory power outside of, the discourses through which these realities are interpreted.

It is because politics is produced through discourse rather than by material conditions that class and economics must forfeit their central importance in Laclau and Mouffe's model. Laclau and Mouffe argue that “workers’ struggles … obviously cannot be explained by an endogenous logic of capitalism,” that divisions between workers are “political, and not merely economic,” and that the very different class configurations and forms of workplace organization across various capitalist countries illustrates that there is no such thing as an “economic” reality prior to politics; the “idea of a workers’ identity around common interests, derived from a class insertion in the relations of production” is therefore false (1985, pp. 80‐82).

Since there is no privileged subject constituted by objective conditions, politics cannot be based on the positive assertion of class—or any other objective—interests. What are required, Laclau and Mouffe argue, are “chains of equivalence” (p. 133) whereby diverse elements unite through negative identification against elites and oppressions; this is a unity that avoids total closure and the erasure of differences, and within which particular political projects maintain their specificity. Laclau and Mouffe suggest that, in the 1980s’ context of an advancing neoliberal project that forces all identities to conform to market logics, a politics of “radical democracy” that seeks the multiplication of political identities, demands, and spaces might provide the basis for powerful chains of equivalence. Aside from their advocacy of radical democracy in the political sphere, Laclau and Mouffe do not advocate particular measures to restructure society and economy; this is unsurprising, since they insist there can be no particular set of interests from which such transformation can properly be deduced.

Laclau and Mouffe's firm rejection of a materialist politics makes their claim to be part of the anticapitalist tradition unconvincing. As Geras (1987) argues in his acerbic critique, Laclau and Mouffe succeed in demonstrating that material conditions are not the sole determinants of political struggle, but not that they are of no relevance. For example, we can consider Laclau and Mouffe's comments on the relationship between workers and their employers. While they correctly note that working conditions vary vastly between and within different countries, that the political arrangements that produce particular working practices are important, and that discourse can make sense of relationships of exploitation in a number of ways (1985, pp. 80–82), they fail, as Geras argues, to demonstrate why, “notwithstanding the wide diversity, a common structural situation, of exploitation, and some common features, like lack of autonomy or interest at work, not to speak of sheer unpleasantness and drudgery” should not be taken as a “solid, objective basis—no more, but equally no less—for a unifying socialist politics” (1987, p. 50). In other words, a broad shared experience of alienation and exploitation is one key element that socialist discourses must connect with and address.

It is important to note that such an overarching socialist critique need not ignore the numerous other forms and sites of subordination, oppression, and antagonism that exist—though Laclau and Mouffe are of course right that some strains of leftwing politics have been guilty of such neglect historically. One notable attempt to reconcile anticapitalist class politics with attempts to redress a multitude of oppressions comes from advocates of social reproduction theory (SRT). SRT argues it is a mistake to treat different forms of discrimination as separate and therefore amenable to different remedies, since the oppression faced by women, racialized minorities, and others is in fact integral to the capitalist system of exploitation as a whole. Unpaid housework carried out by women and poorly paid domestic labor performed by (largely female) migrant workers, for example, aid capitalists by enabling the free or cheap reproduction of labor power. On the basis of this analysis, the working class as a whole—broadly defined—has a strong prima facie interest in abolishing all forms of oppression that contribute to capitalist exploitation (Bhattacharya, 2017).

Laclau and Mouffe's framework sees society as an infinitude of sites of antagonisms and resistance that are irreducible to one another; a socialist, anticapitalist politics, by contrast, conceives of a unified—if heterogeneous—subject whose labor sustains capitalism and which therefore possesses the agency to overthrow it. While Laclau and Mouffe's discourse‐centric model does help us describe some aspects of contemporary radical activist practice, there are others—in particular, the continued appeal of class politics and the significance of the material context in shaping activist strategies—that it cannot account for. Below, I outline my case study, the small activist group Our London, before arguing that while Laclau and Mouffe's model helps us describe aspects of OL’s approach, ultimately their framework cannot adequately explain either Our London's successes or its failures.

## 3 CASE STUDY: OUR LONDON

Our London was founded in January 2015. The group's strategy was to create, using tactics similar to the Spanish “citizens’ platform” Podemos,^1^ a crowd‐sourced “People’s Programme” from demands suggested by thousands of Londoners, and to find “People’s Candidates” for Mayor and Greater London Assembly to put forward this program at the London elections in May 2016. The initial group of around 30 Our London organizers was a mixture of experienced activists working on issues like housing, anti‐racism, and environmentalism, and younger people who had connected with radical politics through either political education workshops for teenagers or through spoken word or other cultural activities. Demographically, there was a rough 50/50 split between those who self‐defined as middle class and had more experience of activism, and black or Asian working‐class young people who tended to have less experience of activism and a much weaker sense of allegiance to “the Left” (see Table A1 in the appendix for details). This political‐demographic balance at the core of the group persisted throughout the entire research period, though the personnel involved gradually changed.

Our London went on to establish itself as a small but significant presence on the Left activist scene in London. From January 2015 onwards, the group held regular meetings and established a core group of activists. Some funding was secured in spring 2015 and used to develop a website, produce a promotional clip, and—in August 2015—appoint a part‐time paid organizer. In October 2015, following the election of left‐winger Jeremy Corbyn as leader of the Labour Party and the selection of Sadiq Khan as Labour's London Mayoral candidate, Our London decided not to stand a candidate for London Mayor nor for the proportionally elected party list element of the Greater London Assembly (GLA). Instead, the group would stand one candidate for a single GLA constituency.^2^ Between May 2015 and March 2016, OL activists ran around 70 People’s Programme workshops with a range of groups including college and university students, migrant workers, Kurdish‐Turkish community organizations, young homeless people, gym workers, community centers, and youth clubs. The research period culminated in an election campaign in which a core group member, Laila, stood and received nearly 1,500 votes.

I define my research method as a critical form of “militant ethnography”: “a politically engaged and collaborative form of participant observation carried out from within rather than outside grassroots movements,” and oriented towards appreciating “the concrete logic of activist practice” (Juris, 2007, pp. 164–165). Given my close involvement with the object of my research, it was also appropriate to incorporate some elements of autoethnography (Denshire, 2014) into my research methods, including personal, subjective analysis of my thoughts, actions, and experiences during the campaign and open discussion of the degree to which I acted upon and influenced the research case study. The aim was to reveal rather than obscure my located role as an activist‐researcher. My role as a founding member of the group studied meant there was, inevitably, a power imbalance between me and my research participants. I attempted to mitigate these by organizing focus groups to allow respondents to question or validate my conclusions, being interviewed by a fellow activist, and sending draft chapters to participants for feedback. Despite these measures, this is no doubt a highly partial and personal study. Data referenced below includes interview transcripts, internal documents, emails, and observation notes. All names used are pseudonyms.

## 4 UNITING THE FRAGMENTS: CHAINS OF EQUIVALENCE IN RADICAL LONDON

Laclau and Mouffe might argue that, in a city like contemporary London, the form of radical unity they propose—that is, a coalitional collectivity that respects the specificity of multiple perspectives, identities, and conceptions of oppression—is the only one viable. If, as Thompson argued in the 1960s, “‘Radical London’ has always been more heterogeneous and fluid in its social and occupational definition than the Midlands or Northern centres grouped around two or three central industries” (1963, p. 23), waves of deindustrialization, financialization and globalization, migration and turnover of population in the nearly six decades since Thompson wrote have further compounded London's diversity to the degree the city is now a byword for social heterogeneity and fragmentation (Massey, 2007). What Thompson described as the need for political “sophistication” in “knitting together” a radical polity in the UK capital (1963, p. 23) is therefore arguably greater than ever.

In some ways, Our London activists recognized this complexity and approached their task as one of building a unity‐in‐diversity from London's fragments. As Table A2 in the appendix shows, OL collectively spoke of multiple subjects, or a plural as opposed to singular subject, and—in line with Laclau and Mouffe's model—referred to a number of different forms of antagonism in descriptions of its constituency and purpose. Some of the categories we used referred to different sections of the population—for example, “riots society” versus “average income families”—while others identified different forms of oppression, like “People of colour, women, the LGBTQ+ community, religious minorities, people with disabilities.”

Another way Our London's political practice seemed to follow Laclau and Mouffe's recommendations was in identifying a common enemy in order to build unity among the disparate groups discussed above. Instead of positively asserting a particular identity that encompasses or overrides all others, Laclau and Mouffe argue that unity is better won through negativity—through highlighting a “constitutive outside” in opposition to which a variety of groups can find common ground and construct “chains of equivalence” between them (1985, pp. 127–134). In line with this logic, OL consistently framed its project as an attempt to dislodge the entrenched power of London's super‐rich and their political clients. For example, the speech Jenny, Will, and I delivered at the first OL meeting in January 2015 outlined the proposed core message of the campaign thus:

Politicians are a corrupt and self‐serving bunch who have repeatedly cheated the people. They have run London for their wealthy parasite friends, and have exploited the people who actually do the work that makes this city profitable and a desirable place to be. (document, January 17, 2015)

This discourse seemed to strike a chord with activists at the core of the group, and was echoed in Our London's public, collaboratively written communications: the “About” page on the OL website identified the adversary as “the super rich and the corrupt politicians who serve them,” and noted that “bankers, billionaires and property developers have bought our politicians, ensuring that mayors, councillors and MPs meant to serve the people of our city instead only look after the interests of the elite” (document, September 3, 2015).

Although we often cast the enemy in purely economic terms, the varied politics, backgrounds, and perspectives at the core of the group meant that OL sometimes highlighted the gendered and racial characteristics of our “constitutive outside.” The preamble to the Our London election manifesto published in April 2016, for example, defined the elite enemy in relation to politics, economics, and race: “the super‐rich and the City of London, career politicians who don't represent us, exploitative landlords and estate agents charging rip‐off fees, councils colluding with property developers in gentrification, a racist, unaccountable police force and big businesses paying poverty wages” (documents, April 28, 2016).

## 5 DECENTRALIZATION AND “OPEN SOURCE ORGANIZING”

Some activists felt that the plural and diverse London subject could only express itself through a pluralist conception of strategy and tactics. These activists supported an “open source organizing” approach bound up with an understanding of oppressions and identities—similar to that expressed by Laclau and Mouffe—as incommensurable and irreducible to one another. From this perspective, OL’s project was one of radical democracy: to carve out numerous political spaces in the city so that groups could express their own—plural, multiple—identities, experiences, interests, and demands.

Calls for a decentralized model were motivated in part by critique of Our London's organizing culture as restrictive of activists’ autonomy. Attempts to secure agreement from a group of activists with varied politics and understandings of strategy had frustrated those whose conceptions lost out, as well as placing huge burdens on weekly organizing meetings; allowing far greater autonomy for sub‐groups of activists was seen as a neat solution. The specific example offered most often in support of the argument for decentralization was a discussion over a proposed event on Islamophobia during the last meeting before Christmas 2015. Nina cited this episode as an example of how meetings could be “disempowering and inefficient” (email, February 13, 2016). The result of the discussion was that Nina's proposal for a Muslim‐led event on Islamophobia—intended to explore the relationships between contemporary Muslim identity and colonial or racializing discourses—was shelved due to disagreements over the ideal audience. A more decentralization model of organizing, it was argued, would remove the need for a single unified conception of our strategic role by allowing sub‐groups of activists to organize however they wished.

The argument for a more decentralized organizing approach had become dominant by the time a February 2016 meeting agreed in principle to adopt Peter's suggestion of what he called the “open‐source organizing” model: any group of activists (including those not previously involved with Our London) could run an event or protest action in the name of OL (notes, February 23, 2016). A shortage of activists and the decision to continue with the mammoth task of fighting the London elections meant little open‐source organizing materialized during the research period. Nevertheless, activists continued to announce their commitment to the spirit of the idea throughout the spring of 2016. Laila, who had previously had mixed feelings on the decentralized model, began to argue for it—for instance, while giving her interpretation of the final weeks of the Ahora Madrid^3^ election campaign she had heard about at a public event: “Citizens had taken the campaign and run with it, expressing it in their way through art, singing, whatever. We need to let go of the brand of Our London” (email, February 22, 2016). Similarly, in April 2016 she told a magazine: “my dream is to have people using OL to run with whatever ideas they want to, as long as they keep our key principles in mind to respect the needs of others” (article, April 15, 2016).

The preference for an open‐source organizing model was associated with a conception of OL’s role as empowering—as deepening democracy by opening up multiple political spaces in the manner proposed by Laclau and Mouffe. Arran, for example, argued that OL’s role was to:

creat[e] spaces for people to speak from their positions of marginality … all these voices, when brought together, create a politics enthused with the struggles and reflections of people’s everyday. … we are merely the vessel through which our audience becomes able to hear itself and act on the reflections that it did not, or had not given itself the time to listen to. Thus, our job is to create spaces in which that reflection is recognized as having power. (email, February 11, 2016)

## 6 CLASS POLITICS IN THE GLOBAL CITY

While some activists emphasized a plural and decentralized conception of Our London's political strategy, others strove to articulate a more strongly class‐based collective subjectivity. Indeed, even some of those activists who argued that the radical London subject was irreducibly plural also spoke at other times in favor of a more unified collectivity. For long periods, particularly at the beginning of the project, the group as a whole seemed eager to move beyond the kind of negative conception of unity Laclau and Mouffe propose—in our case, a unity borne of shared opposition to London's 1%—towards a more positive vision of shared interests and identity. Activists often articulated this stronger conception of unity in terms of our shared experience of being exploited, both for our paid and unpaid labor and for our rents, by bosses, financiers, and property developers. A line—theatrically addressed to the city's exploitative elite—from a poem read out by Laila and Arran on a packed London bus captures this politics: “While you regenerate this city, it seems you've forgotten who generates this city” (clip, May 4, 2016).

One factor that pushed the group to think and act as a unified subject was that, despite some support at times for the “open‐source organizing” model discussed above, the central tactic throughout the project was to develop a People's Programme to contest the London elections. Building a political program meant finding common ground among the activist core in order to develop a shared analysis of exploitation and oppression in London. In practice, this involved incorporating racial and other antagonisms within a broader aggregative frame, as seen in our attempts to link OL’s approach to the housing crisis with race and gender during a speech at a conference in February 2015:

We want to argue that housing is a class issue—it is part of the class structure of London.

And let us stress that by that we don't mean that class is the only basis upon which people in London are suffering over housing—there is a race dimension, in that the culture of gentrification is definitively white and pushing out other cultures, and there is also a gender dimension in terms for example of the disproportionate impact of benefit changes on women.

What we do mean by saying housing is a class issue is that the housing situation in London reflects the balance of forces in this city between 1) economic and political elites, 2) their client group—which is at most 20% of the City's population, and 3) the rest of us—the vast majority (document, February 21, 2016).

Though this kind of framing was broadly popular, there were debates over how our vision of unity should be expressed. Elena, for example, argued that Our London risked ignoring racial oppression in its focus on opposition between “the people” and political‐economic elites: “We all realise how racialized capitalism is and so this should be mentioned and stated in our mission statement” (minutes, May 26, 2015). Elena also suggested that the group's focus on inequality within London itself was problematic, given the capital's exploitation of the global South: “although we're a London centric project, we can't think about London without thinking about its relationship to the rest of the world” (interview, January 16, 2016). As well as strengthening OL’s internal unity, expanding our concept of the London elite to incorporate its involvement in global dynamics of exploitation would have helped develop what Massey (2007) characterizes as a politics of place that “looks from the inside out” (p. 193).

Despite these disagreements, and power imbalances related to race and gender that it is beyond the scope of this essay to discuss, it is nevertheless true to say that the desire to draw together a broad swathe of Londoners, along with our characterization of the elite in class, racial, and gendered terms, facilitated unifying dynamics. The final set of demands published in our program, for example, consisted of a mixture of essentially social‐democratic, class‐based policies like the restoration of the education maintenance allowance (funded through taxation of private schools) and “reclaim[ing] empty properties for social use,” along with demands oriented to racial grievances such as: “Replace the Met … the Metropolitan Police remains a corrupt, unaccountable and racist institution. We need to replace it with an accountable and transparent police force” (document, April 28, 2016).

## 7 “WHERE IS THE WORKING CLASS IN LONDON?”

The unified collective subjectivity glimpsed in the kinds of discourses outlined above was ultimately short lived. This was partly because the growth of the Corbyn project, along with Our London's underwhelming 2016 election result, raised the question of whether it was useful or tenable to keep the project alive. It was also clear that the expressions of collectivity noted above did not solidify into a stable and coherent group socio‐political identity. While our sense of “them”—the elite enemy to be fought—was clear, the identity of “us” was never adequately filled in. We struggled to move beyond what Laclau and Mouffe term “negativity” (1985, p. 129) to develop a more substantive understanding of our own constituency. One element missing from our politics in this respect was a full sense of our own interests, as Oliver reflected:

What I would say is that identity is always the wrong place to start—you always have to start by thinking about interests. Identities only become crystallised when they are effectively expressing a set of objective interests. … So for example I think if you were gonna do this successfully in London you'd have to find a set of the most obviously identifiable interests, and the most obvious one is housing. … I think you would have to take a material issue like housing and make that the basis but then you'd have to create an identity that would invoke a sense of shared investment in that issue and in a project to change it. (interview, June 24, 2016)

While Our London did compile a set of demands, its People's Programme, these were not discussed as emanating from the common interests of the core group of activists itself.

To understand why, we need to move beyond a focus on discourse alone and consider the material context in which Our London attempted to develop a radical collective politics. We found it difficult to articulate a commonality that could overcome social differences and inequalities both within the group and among the broader London working class. Laclau and Mouffe's model would interpret these difficulties mainly as a failure to achieve “relative closure” through discourse: we were unable to settle upon a unifying identity category or master signifier that could have galvanized the chain of equivalence connecting our different subject positions (1985, p. 132). This is one way to *describe* the way our politics developed, or failed to develop, at a discursive level, but it cannot *explain how* the material context of neoliberal London presented obstacles to building collectivity. What makes some discourses resonate while others fail to connect?

Part of the problem for OL was that we were, at times, more keenly aware of differences and inequalities within the group than of our commonalities. Becky and Steph, for example, were the only activists interviewed who were neither graduates nor current university students; they both articulated, in different ways, feelings of class insecurity and were acutely conscious of the differences between themselves and other young activists who had graduated. Steph lamented: “Everyone has a degree now—everyone has a degree … it’s not an unusual thing any more, it’s the norm. So for someone who hasn’t even got a degree, it’s even harder to find something that’s worth doing that’s not gonna kill you coz you’re working 60 hr a week … Even apprenticeships—the amount of money you get paid on apprenticeships is ridiculous – I can’t survive on that money’ (interview, June 20, 2016). Will, moreover, was a graduate and privately educated: he admitted to having “difficulty in a way in fully identifying with the group that I’m talking about when I’m trying to appeal to a working‐class person to get involved. So I feel some discomfort” (interview, July 18, 2015). These perceived internal differences and inequalities were also seen as linked to power imbalances within the group—the case of the shelved public event on Islamophobia (mentioned above) is one example of a conflict bound up with inequalities of power and confidence.

Activists also felt that London beyond the activist core was difficult to conceptualize in class terms, as Jenny reflected:

Like, where is the working class in London? … London’s like a very very mixed city—like how do you get a unified identity when let’s say you’ve moved here from Colombia and let’s say you were really working‐class in Colombia but you’ve come here as a student? And there’s like the international politics of it, there’s race, there’s gender, there’s housing, there’s work, there’s everything. So it’s not a straightforward city, I suppose. (T148: interview, June 14, 2016)

The argument here is that London's complexity and fragmentation made it hard for activists to develop what Jameson would call a “cognitive map” of the “properly unrepresentable totality which is the ensemble of society’s structures as a whole”—that is, to conceive our own collective social location in networks of exploitation, and thus our collective capacity to resist and transform these relations (1991, p. 51).

Another way the social realities of London life presented obstacles to building a unified collectivity was around activists’ divergent preferences on organizing culture and group structure; these dispositions were bound up with activists’ experiences of work. Campaigning around a shared, unified conception of our own class interests and politics required a form of organizing that was unappealing to some, particularly younger, activists, many of whom preferred what was termed the “open‐source organizing” model to practices based on extensive collective deliberation and agreement. Broader contexts of work and activist cultures are important here. Those activists most attracted to a decentralized model of activism based around the metaphor of multiple spaces were creative workers, and it is perhaps testament to the decline of traditional far‐Left parties alongside the rise of social enterprises and the “creative industries” (McRobbie, 2015) that many of the core group of OL organizers initially became involved in activism through music and arts activities. Arran got active in the Black Lives Matter movement via a “poetry nonprofit” while studying in the United States, Elena started to engage with politics through “art interventions” and Nina was first politicized while volunteering on music projects in youth clubs while at university (interviews, January 12 and 16, 2016).

These activists’ conceptions of political organizing seem bound up with a curatorial disposition concerned with producing motivating affects, safeguarding personal creative autonomy and operating within fluid organizational structures. Having rejected as career options both professional activism and academia, Nina explained that she would like to work in “arts spaces”: “Really I’d love to do what I do now, but get paid whilst I do that … If someone paid me to be in [the music studios where I volunteer] for like all my life, that is where I’d be. That place is amazing” (interview, January 12, 2016). While Nina counterposed the alienation she anticipated a campaigning or academic career might involve with the satisfaction and freedom of art, Arran made a similar comparison between art and conventional activism, reflecting that his ideal balance would be “allowing conversations and dialogues from the politics to come into my art but” building his “own medium of expression to a point that I feel comfortable with” (interview, January 16, 2016).

The above comments recall McRobbie’s (2015) stress on the centrality within the discourses, technologies, and practices of “post‐welfare governmentality” of “passionate work”—an “escape from drudgery and monotonous work in favour of self‐directed, and more autonomous activity” (pp. 91–114). In McRobbie's formulation, desires for autonomy and emotional fulfilment from work—and, I would add on the basis of my study, from activism—cannot be understood in merely linguistic or conceptual terms, but rather as subjectivities bound up with the material realities of contemporary capitalism. This illustrates the need to move beyond Laclau and Mouffe's discourse‐centric model in order to understand activists’ politics dialectically—as a product of both ideas and experiences, and the interrelations between the two.

## 8 DISCUSSION AND CONCLUSIONS

Like earlier studies into protest and social movement politics in post‐Fordist societies (e.g. Della Porta, 2005), the findings of my study illustrate how contemporary radical mobilizations tend to be cross‐cut with different political identities, priorities, and strategies. Laclau and Mouffe (1985) argue that political unity can be won in such contexts by building chains of equivalence that unite subjects in shared opposition to, or disidentification from, a common enemy. Theirs is a partial and provisional unity that enables common identification and action but does not collapse multiple identities into one overarching collective subject.

Laclau and Mouffe's framework does capture elements of how Our London organized: in particular, our focus on identifying a common enemy as a way of uniting participants and our emphasis on multiplicity alongside unity are broadly in line with Laclau and Mouffe's recommendations. Furthermore, this study casts light on the implications for group structure of organizing on the basis of a plural and provisional unity of the type Laclau and Mouffe describe. What OL activists called the “open‐source organizing” model was in some ways aligned with an emphasis on unity‐through‐negativity as opposed to a more totalizing form of collective subjectivity. It is also true, however, that some activists saw our failure to develop a stronger form of unified collective subjectivity to be more of a weakness than a strength, and the group’s trajectory—including our difficulties in articulating a sense of common collective interests—suggests they may have been right.

While my study partially validates some aspects of Laclau and Mouffe's arguments, it also points to clear shortcomings in their framework. At the heart of these weaknesses is the fact that their model—because it denies that material conditions determine politics—cannot explain why class politics is centrally important, and often has a particularly potent appeal, in capitalist societies. Discourses that speak to economic interests and address the realities of economic exploitation are not important simply because they connect with identities, but because they resonate with our experiences and understandings of our social system. It is partly for this reason that growing inequality, social dislocation, and the implementation of austerity in the years following the 2008 financial crisis have given rise to a new wave of class politics. Recent left‐wing electoral projects—such as Corbyn's Labour in the UK, Bernie Sanders’ primary campaigns in the US, Sinn Fein in Ireland and Podemos in Spain—have placed criticism of austerity, privatization, tax avoidance, property speculation, and other features of neoliberal capitalism at the center of their politics. Our London activists similarly developed a critique of rentier capitalism in London as part of their efforts to go beyond a purely negative unity‐in‐opposition and develop a sense of commonality based on our common experience of exploitation. Borrowing Jameson’s (1991) terminology, I argue that OL’s denunciation of extractive industries like finance and property development helped to develop a “cognitive map” of our common social location and political agency within the city (p. 51).

Laclau and Mouffe's model cannot adequately explain the appeal of such discourses: they criticize the conventional Marxist tendency to center class exploitation on the dual grounds that, first, subjects are constituted by a multiplicity of overlapping oppressions rather than simply one master‐dynamic and, secondly, that attempts to subject every social and political antagonism to a crude class analysis will likely alienate groups who define their struggle in other terms. My study conversely demonstrates that a class‐centric politics (albeit one which does not deny the significance of other forms of oppression) can play a powerful unifying role. Indeed, as Oliver suggested, if the group had developed the materialist aspects of its politics further and spoken more clearly about shared interests, this could have made the project stronger and more sustainable. To use Gramsci’s (1971) terminology, I argue that OL activists struggled to attain “consciousness … of the solidarity of [class] interests among all the members” (pp. 181–182) of our group of relatively young cognitive laborers, and that this difficulty was key to the project's weaknesses.

My study also confirms Riofrancos’ (2018) emphasis on the importance of analyzing “the gritty politics and the exigencies of organizing” and her criticisms of Laclau and Mouffe's discourse‐centric model for neglecting these. I argued above that some activists’ attitudes towards group structure, internal democracy, and organizing culture—features I discussed as part of a “curatorial disposition”—must be understood in the context of contemporary capitalist cultures that value networked autonomy and fluidity, and within which younger activists in particular were deeply embedded. That activists’ working lives shaped their approaches to political activity again highlights the deficiencies of a model like Laclau and Mouffe's that focuses on discourse and insists that material conditions do not determine politics. We can instead agree that politics is not a product of economics, but argue that the latter does nevertheless play a role in “the setting of limits” for and “exertion of pressure” on the former (Williams, 2005 [1980], p. 34).

Laclau and Mouffe highlight important aspects of how collectivity and unity can be built in conditions of social fragmentation. However, my study suggests that a focus on discourse alone is not enough. To fully appreciate the dynamics involved in contemporary radical Left politics, we must consider the significance of objective interests, the dispositions and social locations of grassroots activists, and material changes in relations of exploitation that have opened the possibility for new political currents to develop—all aspects for which we will have to look well beyond Laclau and Mouffe's framework.

## Data Availability

The data that support the findings of this study are available on request from the corresponding author, Jacob Mukherjee. The data are not publicly available due to the need to safeguard confidentiality of research subjects.

## NOTES

^1^
Podemos was a new political party formed in January 2014. It used a combination of digital democracy, social movement mobilization, and charismatic leadership to channel support for the huge Spanish anti‐austerity and pro‐democracy movements into the electoral arena (Fenton, 2016).

^2^
The Greater London Assembly provides minimal checks on the Mayor of London. Its 25 members are elected by a mixed voting system, with London organized into 14 huge constituencies with one representative each and the remainder proportionally allocated; it was for one of these constituency elections that Our London stood (https://www.london.gov.uk/about‐us/london‐assembly/about‐london‐assembly).

^3^
Ahora Madrid was, and is, a radical municipalist group and political party operating in the Spanish capital.

## APPENDIX 1

**Table A1:** Our London activists’ social backgrounds

Name^a^	Age	Ethnicity (self‐described)	Class (self‐described)	Employment	Housing	Education
Antonio	20	Afro‐European	Working class	Student/ retail	Social rent	BA (studying)
Arran	23	Mixed (Caribbean/ white)	Working class	Retail/ poetry/ photography	Social rent	BA (achieved)
Becky	22	White	Working class	Bar work	Private rent	A Level (achieved)
Elena	23	Armenian‐Iranian	Working class	Freelance artist	Social rent	BA (achieved)
Laila	25	Mixed (black/ Chinese)	Working class	Freelance music teacher	Private rent	BA (achieved)
Nina	21	Algerian	Working class	Student/ community work	Social rent	BA (studying)
Ryan	23	Mixed (Caribbean/ white)	Working class	Temporary campaign work	Private rent	BA (achieved)
Steph	22	White	Working class	Electrical engineer	Private rent	A Level (achieved)
Jacob	34	Mixed (Asian/ white)	Middle class	PhD student/ hourly paid lecturer	Private rent	PhD (studying)
Jenny	27	White	Middle class	Teacher	Private rent	MA (achieved)
Josh	25	White	Middle class	Researcher	Private rent	MA (achieved)
Matt	36	White	Middle class	Fitness instructor	Private rent	PhD (achieved)
Oliver	44	White	Middle class	Academic	Owner	PhD (achieved)
Peter	29	White	Middle class	PhD student	Private rent	PhD (studying)
Will	34	White	Middle class	NGO campaigner	Private rent	BA (achieved)

^a^ All names are pseudonyms.

**Table A2:** Selection of terms used to describe the Our London subject

Term [and activist^a^]	Format	Date
“Riots society” [Jenny]	Email to Jacob, Will	November 24, 2014
“Young people at schools, universities and colleges; precarious and migrant workers; people fighting attacks on their social housing and their services” [Jenny, Jacob, Will]	Speech at first OL meeting	January 17, 2015
“Average income families”	Blog post for sympathetic platform	August 17, 2015
“A growing group of disillusioned Londoners”	OL website “about” page	September 3, 2015
“The rest of us” [not “ultra wealthy global property investors”]	OL website “about” page	September 3, 2015
“From homeless young people, to cleaners and Kurdish youth”	OL website “about” page	September 3, 2015
“STUDENTS// PERFORMERS// ACTIVISTS// COMMUNITIES// WORKERS// MIGRANTS// LONDONERS” [Laila + Arran]	Election leaflet #1	April 9, 2016
“The people”	Election manifesto	April 28, 2016
“People of colour, women, the LGBTQ + community, religious minorities, people with disabilities”	Election manifesto	April 28, 2016

^a^ Where activist not stated, phrase is from collectively agreed document.
